# Kelvin probe force microscopy of nanocrystalline TiO_2_ photoelectrodes

**DOI:** 10.3762/bjnano.4.49

**Published:** 2013-07-01

**Authors:** Alex Henning, Gino Günzburger, Res Jöhr, Yossi Rosenwaks, Biljana Bozic-Weber, Catherine E Housecroft, Edwin C Constable, Ernst Meyer, Thilo Glatzel

**Affiliations:** 1 Department of Physics, University of Basel, Klingelbergstrasse 82 CH4056, Switzerland; 2 School of Electrical Engineering, Faculty of Engineering, Tel-Aviv University, Ramat-Aviv 69978, Israel; 3 Department of Chemistry, University of Basel, Spitalstrasse 51 CH4056, Switzerland

**Keywords:** atomic force microscopy (AFM), dye-sensitized solar cells (DSC), Kelvin probe force microscopy (KPFM), surface photovoltage (SPV), titanium dioxide (TiO_2_)

## Abstract

Dye-sensitized solar cells (DSCs) provide a promising third-generation photovoltaic concept based on the spectral sensitization of a wide-bandgap metal oxide. Although the nanocrystalline TiO_2_ photoelectrode of a DSC consists of sintered nanoparticles, there are few studies on the nanoscale properties. We focus on the microscopic work function and surface photovoltage (SPV) determination of TiO_2_ photoelectrodes using Kelvin probe force microscopy in combination with a tunable illumination system. A comparison of the surface potentials for TiO_2_ photoelectrodes sensitized with two different dyes, i.e., the standard dye N719 and a copper(I) bis(imine) complex, reveals an inverse orientation of the surface dipole. A higher surface potential was determined for an N719 photoelectrode. The surface potential increase due to the surface dipole correlates with a higher DSC performance. Concluding from this, microscopic surface potential variations, attributed to the complex nanostructure of the photoelectrode, influence the DSC performance. For both bare and sensitized TiO_2_ photoelectrodes, the measurements reveal microscopic inhomogeneities of more than 100 mV in the work function and show recombination time differences at different locations. The bandgap of 3.2 eV, determined by SPV spectroscopy, remained constant throughout the TiO_2_ layer. The effect of the built-in potential on the DSC performance at the TiO_2_/SnO_2_:F interface, investigated on a nanometer scale by KPFM measurements under visible light illumination, has not been resolved so far.

## Introduction

**Figure 1:**
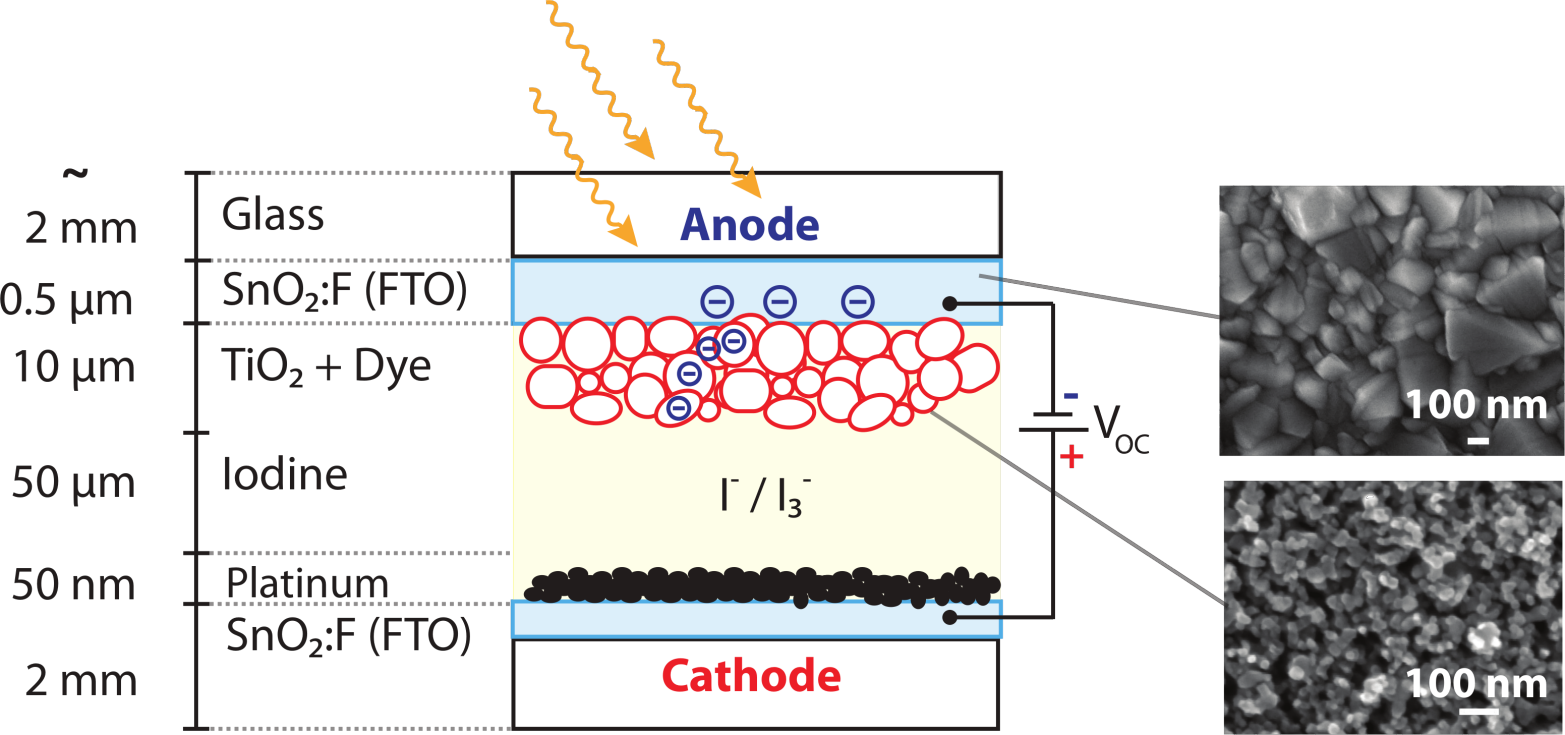
Different components of a DSC under illumination in an open circuit. Upon light excitation electrons are injected from the adsorbed dye molecules into the conduction band, *E*_cb_, of the wide-bandgap metal oxide (nanoporous TiO_2_) resulting in an open-circuit voltage, *V*_oc_.

Dye-sensitized solar cells (DSCs) provide a promising low-cost, high-efficiency third-generation photovoltaic concept based on the spectral sensitization of a nanoporous wide bandgap semiconductor [1–2]. In the past two decades DSCs have received substantial attention from both academic and industrial communities focusing on new materials and advanced device concepts [3–8]. A typical DSC consists of a dye-coated TiO_2_ photoelectrode, deposited on a fluorine-doped tin oxide (FTO) conductive-glass substrate, an 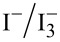 redox-couple-based electrolyte and a platinum counter electrode as depicted in Figure 1. Upon visible-light excitation, dye molecules inject electrons into the conduction band, *E*_cb_, of the semiconductor; the oxidized dye is subsequently reduced by the redox couple of the surrounding electrolyte. The generated electrons diffuse toward the SnO_2_:F substrate and establish the photovoltage. The most frequently used dye complexes contain less-abundant transition metal elements such as ruthenium. Complexes of earth-abundant metals such as zinc and copper are candidates to replace the more expensive ruthenium dyes [9–13]. Recently, Yella et al. reported an efficiency of over 12% with a porphyrin-sensitized DSC and a cobalt(II/III) based redox electrolyte [14]. However, many details of the hybrid organic/inorganic interface and the influence of subsequent preparation steps on the device properties, e.g., surface topography and potential, are still unclear and have the potential to increase the efficiency and long-term stability of the devices. Investigations of nanoscaled photovoltaic devices require nanometer-scale measuring methods, including time-resolved measurements of the carrier dynamics [15–16]. Although a DSC photoelectrode consists of a nanostructured TiO_2_, there are few microscopic studies [17].

**Figure 2:**
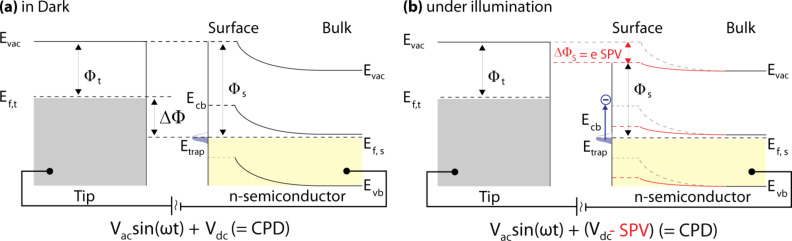
Schematic band diagram for a KPFM tip in close proximity to an n-type semiconductor surface (a) in the dark and (b) under illumination while the CPD is nullified by an applied dc voltage. Upon illumination the local vacuum energy level, *E*_vac_, is shifted downwards and detected as a work function or CPD decrease, which results in a “negative” SPV. *E*_f,t_ and *E*_f,s_ are the Fermi levels of tip and sample, respectively. *E*_vb_ and *E*_cb_ are the valence and conduction band edges of the semiconductor. *E*_vac_ is the local vacuum and *E*_trap_ a surface-state energy level, respectively. The work-function shift, ΔΦ_s_, upon illumination is equal to e SPV.

Surface photovoltage (SPV) spectroscopy is a non-destructive and sensitive method for determining surface potential changes upon illumination, identifying surface states, and extracting material parameters, in particular the bandgap, *E*_g_, the minority carrier diffusion length, *L*_n_, and the flatband potential, *V*_fb_ [18]. SPV spectroscopy is usually performed with a macroscopic vibrating capacitor and is hence limited by its poor lateral resolution [19–20]. Bare and dye-sensitized nanocrystalline (nc) TiO_2_ have been investigated with such a macroscopic Kelvin probe (KP) revealing details about the electronic structure [21–23], trap states [24], the surface dipole [25], charge-carrier dynamics [26], and indicating changes upon chemical treatments [24,27–29]. KP studies have helped to select surface treatments that are beneficial for the DSC performance. In order to achieve a nanometer scale resolution, SPV spectroscopy can be combined with Kelvin probe force microscopy (KPFM) [30–32], an atomic force microscopy (AFM) technique that was introduced in 1991 [33]. KPFM is a surface-potential detection method that determines the contact potential difference (CPD) during scanning by compensating the electrostatic forces between a microscopic tip and the sample [34]. Figure 2a illustrates a schematic band diagram for a KPFM tip in close proximity to a semiconductor sample surface with surface states, *E*_trap_. An applied dc voltage, *V*_dc_ = CPD, nullifies the work-function difference, ΔΦ, between both materials. The occupied surface states of the n-type semiconductor, depicted in Figure 2a, are depopulated upon illumination with an appropriate light energy. Consequently, the surface band bending of an n-type semiconductor is shifted downwards and the measured CPD decrease is equal to the SPV.

**Figure 3:**
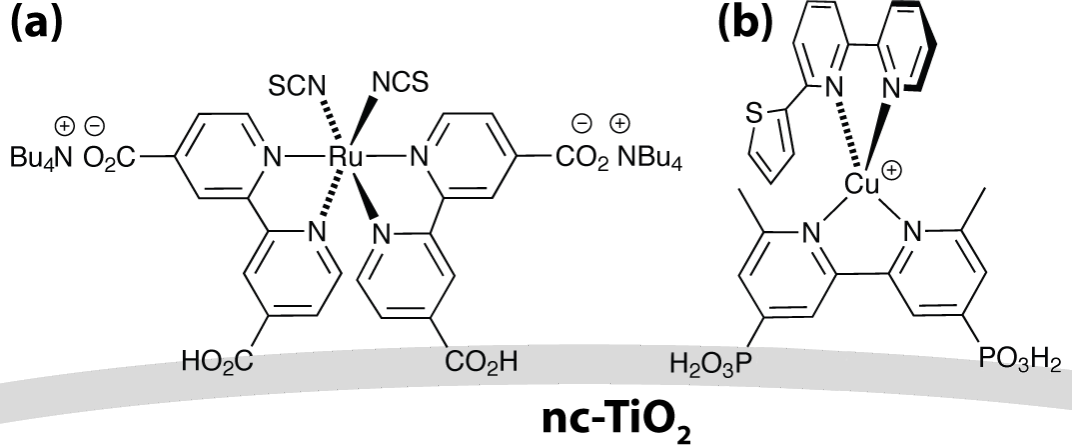
Schematic structures of (a) the standard dye N719 and (b) a copper(I)-based dye, assembled in situ (see text).

The considerably high performance in DSCs is achieved also due to the high surface-to-volume ratio of nanocrystalline TiO_2_. In any case, there is a trade-off between a high surface-to-volume ratio and the carrier transport. Smaller TiO_2_ particles lead to an increase of grain boundaries and reduce the solar cell current. Hence, we have considered it as relevant to characterize the surface potential of nanostructured TiO_2_ with a high-resolution method. Surface dipole changes upon dye adsorption induce a shift of the surface potential in the order of hundreds of millivolts, which is detectable by KPFM on the nanometer scale [35–37]. A direct influence of the surface dipole on the open-circuit voltage, *V*_oc_, of a DSC was predicted by Angelis et al. [38] and experimentally addressed by KPFM investigations [39–40]. KPFM studies in UHV conditions of rutile TiO_2_ decorated with either nanometer-sized Pt clusters [41] or single dye molecules [42] revealed a significant impact of single particles on the surface dipole. We have investigated the surface parameters of DSC photoelectrodes on the nanoscale using KPFM, which is not possible to achieve with a macroscopic KP. SPV spectra were taken on desired locations with a lateral resolution of 25 nm. Thus, the bandgap and time constants were obtained on the nanoscale. In this work, microscopic variations of the work function were observed for both sensitized and bare nc-TiO_2_. To correlate the microscopic changes on a dry photoelectrode with the macroscopic DSC parameters, local surface dipole variations for a ruthenium(II)- and a copper(I)-based dye were determined. The ruthenium(II) dye chosen was the standard dye N719. The copper(I)-based dye (Figure 3) was selected from a range of complexes that we have recently prepared and screened for their potential use as sensitizers [43].

## Results and Discussion

### Work function inhomogeneities

**Figure 4:**
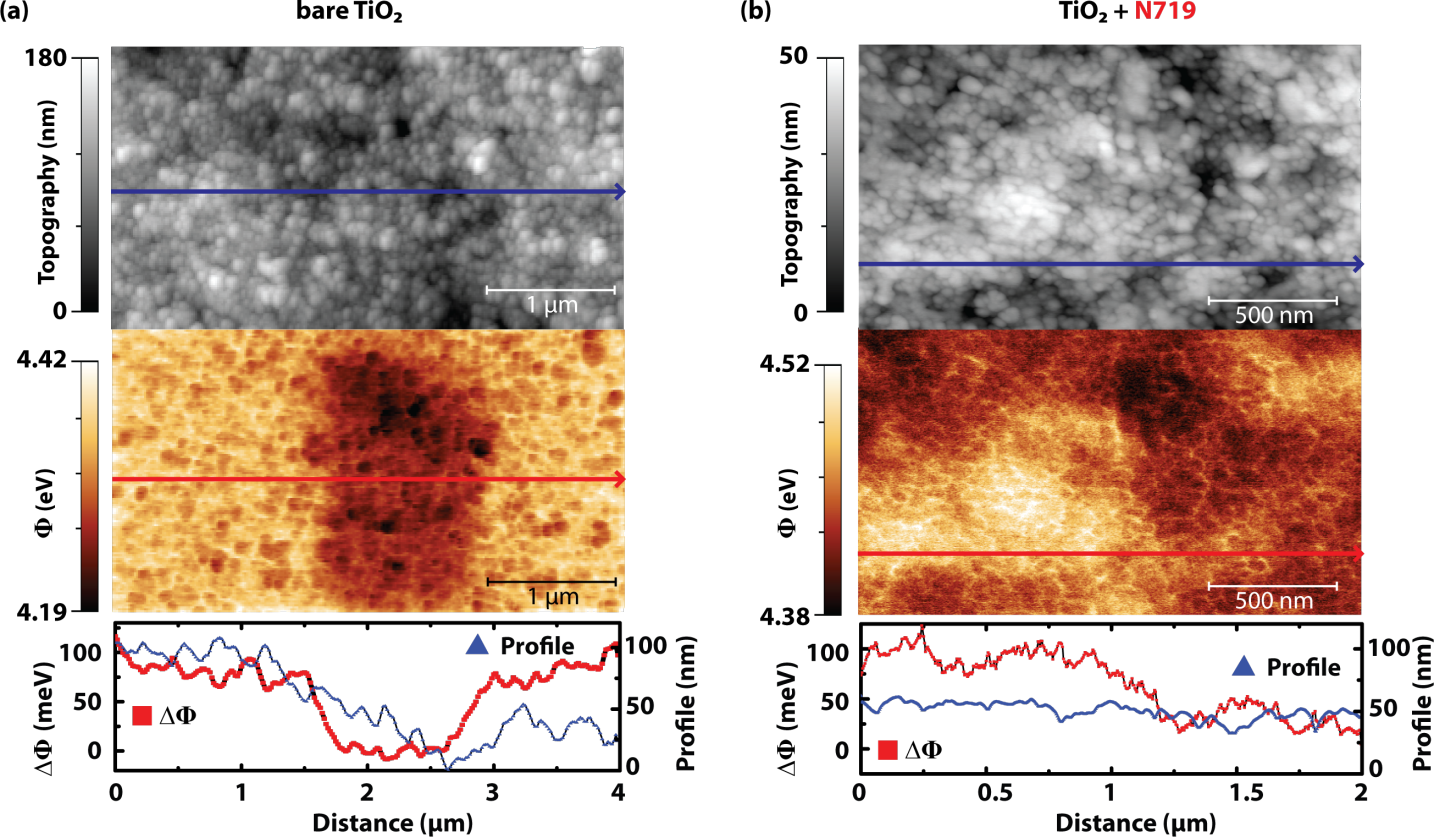
Topography and work function of (a) a bare TiO_2_ and (b) an N719 sensitized TiO_2_ layer with a thickness of ≈10 μm revealing wide-spread inhomogeneities in the work function. The measurements correspond to a scan size of (a) 2 × 4 μm and (b) 1 × 2 μm. Imaging parameters: *A*_free_ = 20 nm rms, *A*_set_ = 70%, *f*_1st_ = 72 kHz, *f*_2nd_ = 452 kHz, *U*_ac_ = 2 V, *T* = rt. The TiO_2_ is a commercial product from Solaronix, Ti-Nanoxide T.

Figure 4 shows the topography and the work function of a bare TiO_2_ and an N719-sensitized TiO_2_ layer measured by KPFM in a dry nitrogen glove box at room temperature. The topography images reveal, in both cases, a homogeneous surface with nanoparticles, nominal diameter of 20 nm, in the range of 20–100 nm. Work-function (Φ) variations reflecting the position of the conduction band edge *E*_cb_ of 80 mV on average, appear for both samples and are visible as dark regions in the measurements. They are highlighted in the cross sections in the lower part of the image. Such a local work function shift can be attributed to local variations of chemisorbed contaminants resulting in a decrease of the local vacuum energy, *E*_vac_, and the electron affinity, χ. A thin water layer consisting of chemisorbed and physisorbed H_2_O molecules on the nc-TiO_2_ is known to be present even inside a dry nitrogen glove box [44]. Solvent residues are further possible contaminants that can be locally attached to the TiO_2_ surface, or the variations may be due to varying material properties in general. In any case, such variations, which are clearly detectable by KPFM, may obstruct the optimal attachment of dye molecules and thus reduce the solar cell performance [25].

### Microscopic surface photovoltage

By combining a tunable illumination system with KPFM, the surface photovoltage (SPV) can be measured on the nanometer scale and is referred to as microscopic SPV. A microscopic SPV is caused by an electron generation upon light absorption at either the surface space-charge region or at the electric field of a buried interface that is reached by the incident light [19]. In the present work, the sample was illuminated with focused light from an optical fiber or directly with a laser. The measured SPV can have two contributions, one from the TiO_2_/SnO_2_:F interface and the other from the TiO_2_ layer depending on the energy of the incident light, i.e., super- or sub-bandgap(TiO_2_) illumination. Both SPV effects are described separately in the following two sections. Time-resolved SPV measurements provide insights into charge carrier dynamics [45] and are described below.

#### Surface photovoltage under super-bandgap illumination

**Figure 5:**
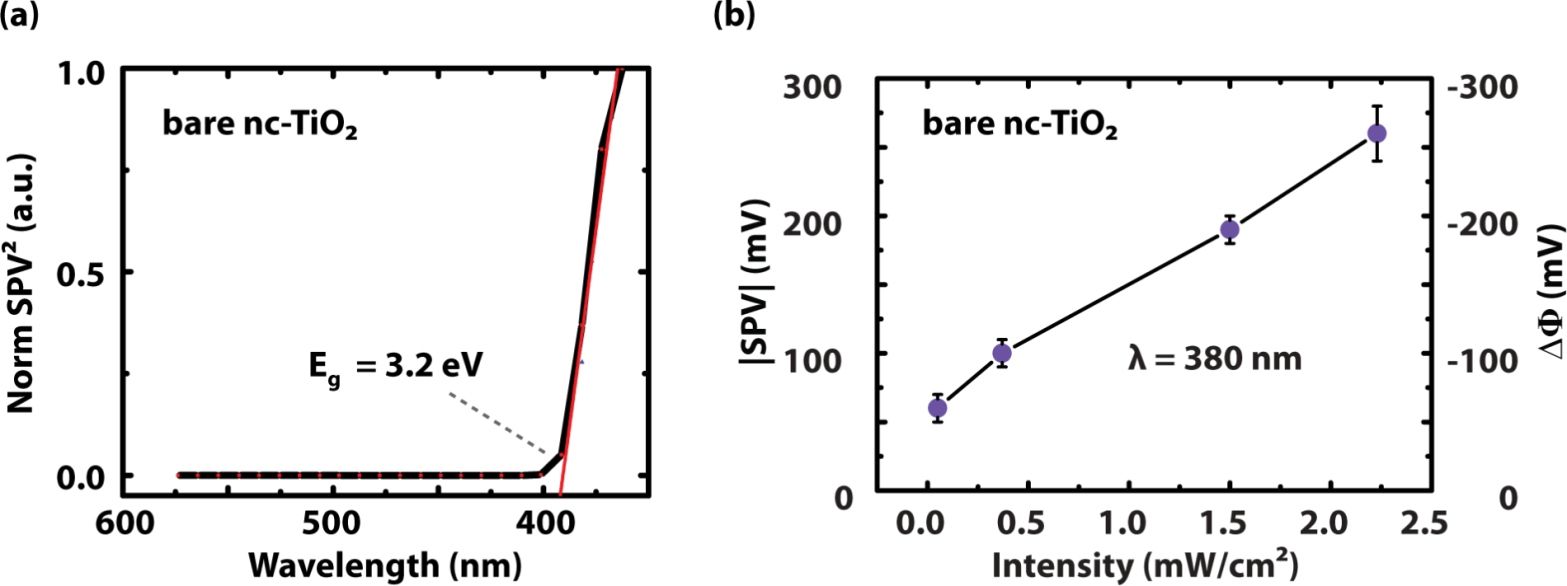
SPV for bare nc-TiO_2_ in dependency on (a) the wavelength and (b) on the light intensity under super-bandgap illumination (380 nm). The bandgap of the material was extracted by SPV spectroscopy.

SPV spectroscopy (SPS) is a common method for measuring the bandgap, *E*_g_, of a semiconductor by determining its dependency on the absorption coefficient, *α*. The obtained bandgap for nc-TiO_2_ (Figure 5a) is in accordance with the literature value for bulk TiO_2_, *E*_g_ = 3.2 eV [46] and validates the SPS setup. The extraction of *E*_g_ by means of SPS is superior to the usual transmission spectra since it is also applicable to thin layers, nanowires, or single nanoparticles and also for opaque samples [18]. Under illumination with a sufficiently low light intensity, the SPV can be assumed to be proportional to the absorption coefficient, implying a maximum SPV for super-bandgap illumination. Depending on the bandgap type, either direct or indirect, the SPV curve is fitted with the corresponding relation [18,47]:

[1]
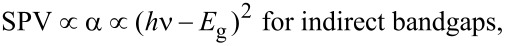


[2]
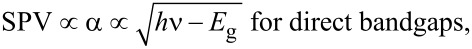


where *h* is the Planck constant and *ν* is the frequency of the light. For anatase TiO_2_, an indirect bandgap material [48], *α* is therefore expected to show a quadratic dependence on the illumination wavelength for energies just above the bandgap. Figure 5a presents an SPS measurement taken on a cluster of sintered anatase particles showing a quadratic dependence on the wavelength. By linear fitting, a bandgap energy of *E*_g_ = 3.2 eV was extracted using Equation 1, assuming a phonon energy *E*_p_ ≈ 0.

Figure 5b depicts the SPV of bare TiO_2_ as a function of the light intensity for super-bandgap illumination with a wavelength of 380 nm. The negative SPV indicates an n-type behavior of the material. The SPV exhibits a linear dependency on the light intensity up to a value of −250 mV. A logarithmic dependence on the light intensity would be typical for a charge separation at a built-in potential, for instance at the surface space-charge region. However, the linear dependence indicates a charge separation, which is governed mainly by diffusion and not by drift current (electric field). Preferential trapping of electrons (holes) in defect states of the TiO_2_ network leads to different diffusion coefficients for electrons and holes.

#### Surface photovoltage under sub-bandgap illumination

**Figure 6:**
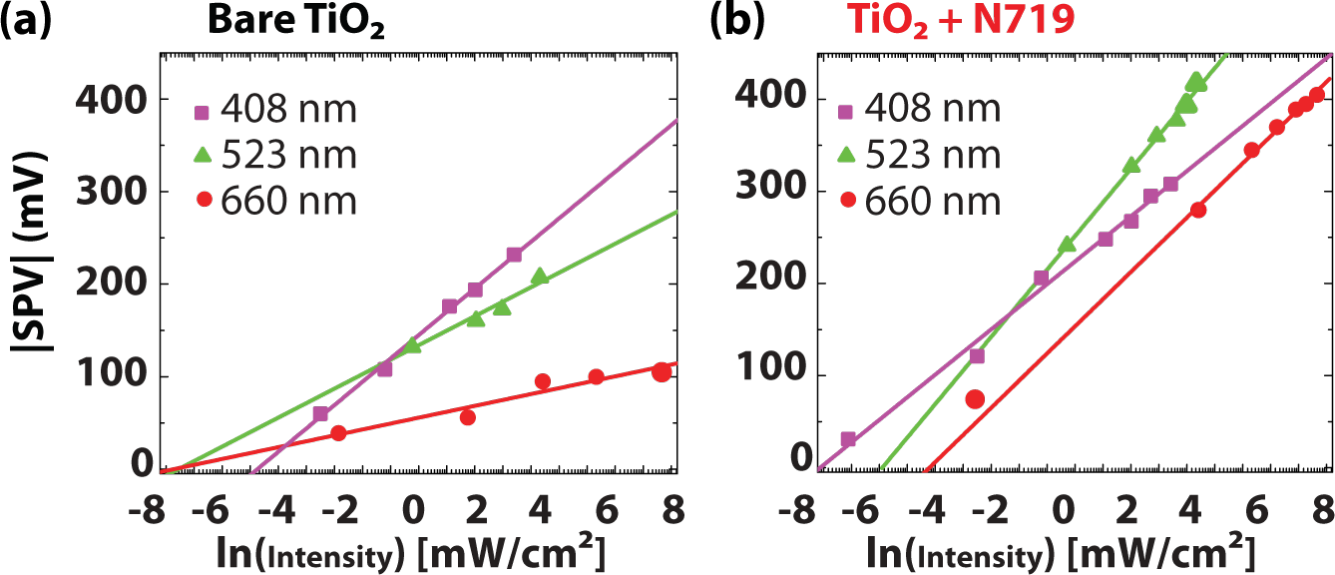
Semilogarithmic plot of the SPV dependence on the incident light intensity, (a) measured for three different wavelengths on bare and (b) N719-sensitized TiO_2_.

Figure 6a shows a semilogarithmic plot of the SPV versus the light intensity for three different wavelengths for the bare nc-TiO_2_. An SPV of −230 mV was reached under sub-bandgap (λ = 408 nm) illumination. A negligible sub-bandgap SPV of less than 20 mV was measured for λ = 408 nm on TiO_2_ layers directly deposited on glass. We assume that the SPV under sub-bandgap illumination results from a reduction of the built-in potential, *V*_bi_, at the TiO_2_/SnO_2_-interface. This built-in electric field is screened by the photogenerated charge carriers resulting in a downward band bending of the TiO_2_ conduction band edge *E*_cb_.

The measured SPV of −250 mV under super-bandgap illumination (Figure 5b) provides an estimation for this downward band bending of *E*_cb_. Figure 5b shows the CPD decrease with onset illumination, which decreases further with higher illumination intensities. A CPD decrease is equivalent to a work function decrease of the TiO_2_. The observed logarithmic dependence demonstrates a photodiode behavior according to Equation 3 and is an indication for a built-in (Schottky barrier) potential at the interface. It should be noted that the photocurrent, *J*_ph_, in Equation 3 is approximately proportional to the light intensity, *I*. When the incident light wavelength approaches the bandgap energy of TiO_2_ higher SPVs result leading to steeper slopes of the SPV-versus-intensity curves. The SPV is proportional to the number of photogenerated charge carriers. It is evident from Figure 6a that more electrons are generated with higher illumination energies within the TiO_2_ network. We assume that empty surface states just below the conduction band edge are occupied by valence band electrons. According to Howe et al. there are localized Ti^3+^(3d) trap states just below the conduction band edge of nc-TiO_2_ [49]. According to our measurements, the SPV decreases exponentially with decreasing illumination energies and we conclude, therefore, that the number of trap states also decreases exponentially with decreasing trap state energy relative to the conduction band edge of the TiO_2_ nanoparticles.

The buried TiO_2_/SnO_2_-interface is reached by the incident light since the nanoporous TiO_2_, deposited on top of the FTO-layer, is only about 10 μm thin and transparent to visible light (*E*_g_ = 3.2 eV). Due to the high n-dopant density of SnO_2_:F (*N*_D_ ~ 10^20^), it can be approximated to being nearly metallic. Generally, the SnO_2_:F contact is regarded as Ohmic since electrons may tunnel through the ca. 2 nm thin barrier at the interface space charge region [50]. However, the TiO_2_/SnO_2_:F contact forms a heterojunction between two wide-bandgap semiconductors, degenerately doped SnO_2_ and (intrinsic) nc-TiO_2_. Kron et al. and Levy et al. [51–52] investigated alternative materials to SnO_2_:F and found that the built-in voltage at the interface has no significant influence on *V*_oc_ of the DSC but does influence the fill factor, FF. With the determined work functions of 4.3 ± 0.1 eV for nc-TiO_2_ and 4.7 ± 0.1 eV for SnO_2_:F in our KPFM measurements, the band offset at the heterojunction allows an estimation of the energy barrier. Depending on the front electrode material, this energy barrier varies and consequently the interface contact resistance differs. As a result, the FF of the DSC can be increased with a lower interface energy barrier and a narrower space-charge region decreasing the sheet resistance at the interface.

For the sensitized TiO_2_, the SPV also shows a logarithmic dependence on the light intensity (Figure 6b). We conclude that the measured SPV of the sensitized TiO_2_ is created by two different effects: a change of the surface dipole (after electron donation) and a charge carrier concentration gradient between the illuminated surface and the bulk due to different diffusion coefficients for electrons and holes (photo-Dember effect). The latter effect causes a potential drop forming an electric field in the z-direction across the TiO_2_ layer [29,53–54]. The Dember photovoltage is caused by a non-uniform generation or recombination of charge carriers within the sample [18]. The adsorbed dye molecules are considered as n-type “photodoping” since electrons are generated under sub-bandgap illumination. After electron injection into the conduction band of TiO_2_, the dye is oxidized and charged more positive relative to its ground state. Hence, the surface dipole is reduced and detected as a change in the CPD.

#### Time evolution of the SPV

**Figure 7:**
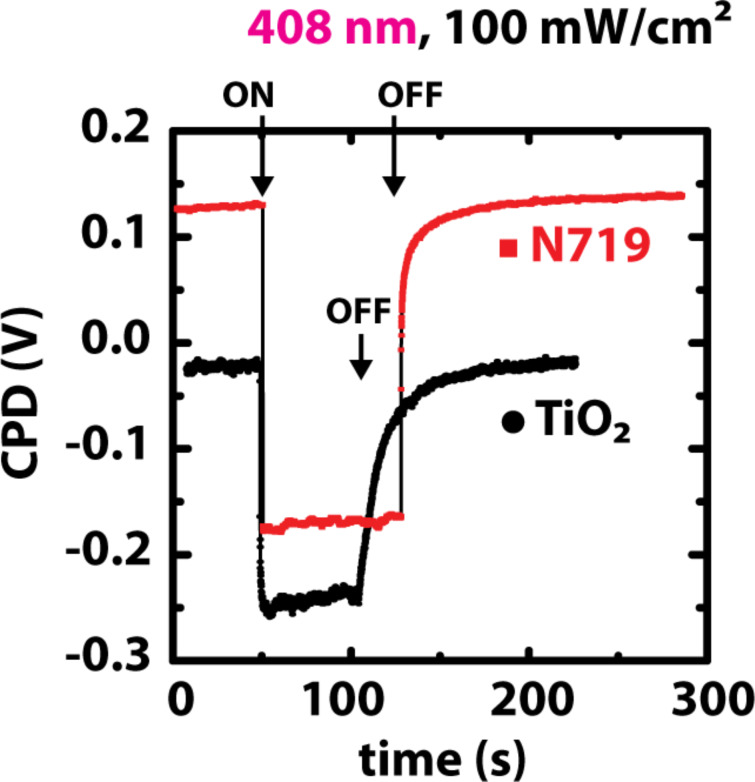
Time evolution of the measured CPD of TiO_2_ and TiO_2_ + N719 during the turning on and off of the violet (408 nm) laser light.

Time-dependent SPV measurements were performed at specific positions above single TiO_2_ particles. Since KPFM is sensitive to potential drops in the entire sample, the measurements give insights into charge-carrier transport processes of the particle network. Figure 7 shows the time evolution of the measured CPD values after the turning on and off of the laser light illumination with a wavelength of 408 nm. For both sensitized and bare TiO_2_ the SPV with onset illumination, *t*_on_, is below the resolution limit (50 ms) of the measurement system. The photoresponse time corresponds to the required time for the charge carriers to reach a steady-state condition upon illumination. In turn the recombination time, *t*_off_, is the time required to reach the initial value in the dark. Recombination times of 65 ± 6 s for bare TiO_2_ and 43 ± 4 s for N719 were determined with KPFM. The recombination curve was divided into a fast and a slow component and approximated as the sum of two exponential functions. The slow component of the total recombination time is attributed to an electron diffusion process across the TiO_2_ network towards the contact, whereas the fast recombination process occurs within single particles [55]. The slow electron diffusion throughout the network is due to trapping and detrapping [46] in surface and bulk defect states. TiO_2_ is regarded as an insulator with a relative permittivity of ε_r_ = 36 and consequently acts as a charge storage capacitor between a metallic tip and a highly conductive SnO_2_:F contact. Upon photoelectric charge injection, the redistribution of charge carriers (by diffusion through the network) is slow (seconds to minutes). A slow response time has also been reported for nanoporous TiO_2_ [56–57] and for porous Si, which exhibited recombination times of up to 1 h [58].

### Microscopic surface-dipole variations

**Figure 8:**
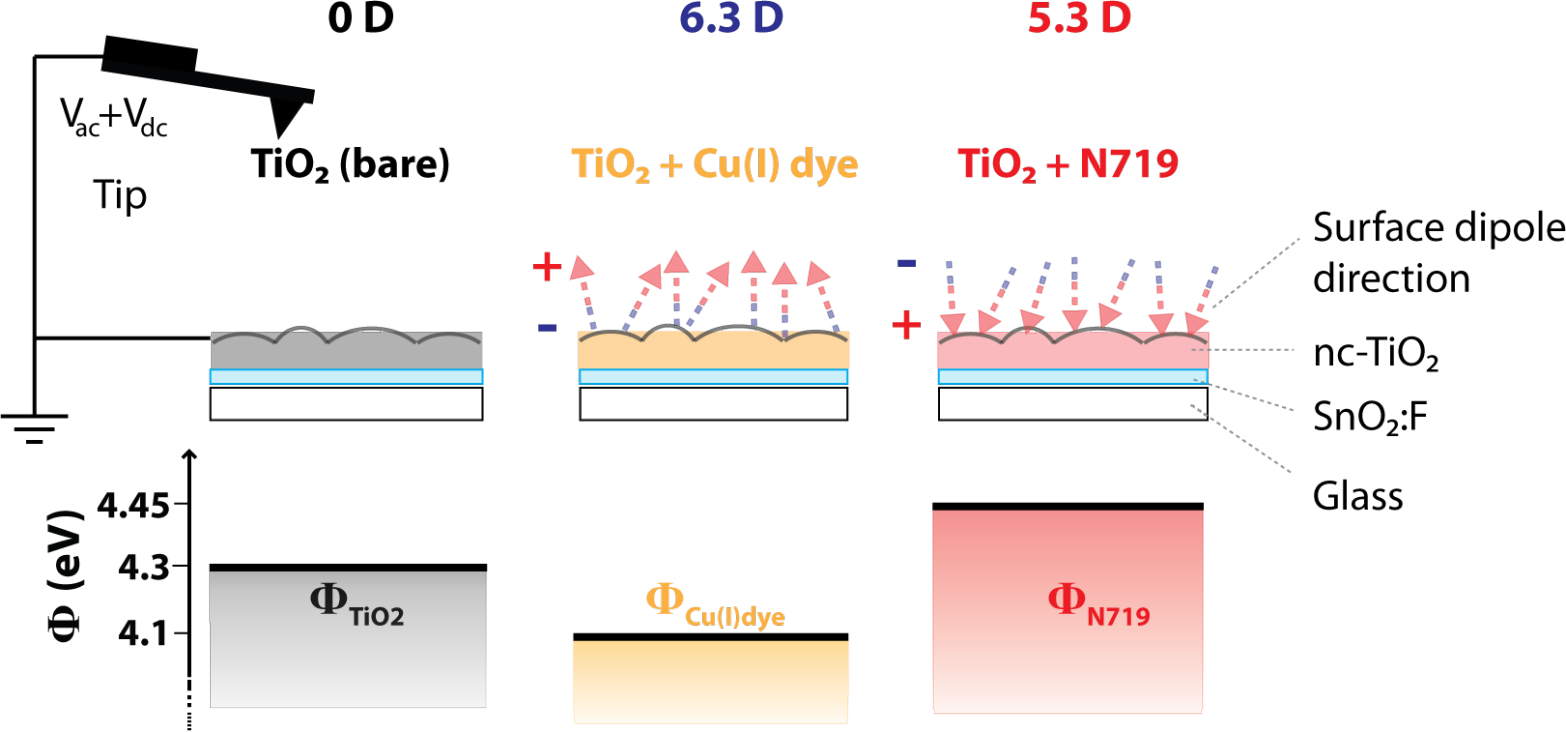
Schematic illustration of the KPFM measurement system and the surface dipole induced by adsorption of the ruthenium containing dye N719 (6.3 D) and the copper(I) dye (5.3 D) pointing in opposite directions. The measured work-function values are compared with the bare TiO_2_ substrate without external illumination.

By averaging the work function values over several images on different sample spots an increase of ΔΦ = 150 ± 40 mV for N719 and an average decrease of ΔΦ = −180 ± 40 mV for the copper-containing dye was determined on sensitized TiO_2_ films by KPFM. The values as well as a model describing the dipole moment strength and orientation are presented in Figure 8.

The surface dipole is the result of a combined effect of both anchoring domain and dye molecule. The effective electron affinity, χ*, is affected by the surface dipole formed by adsorbed dye molecules [59]. The detailed anchoring mechanism for the N719 dye on TiO_2_ is still under debate. It is widely accepted that the N719 is chemisorbed either through two or three carboxylic acid groups. Results by Lee et al. support that an additional hydrogen bonding (physisorption) is present [60]. Molecular dynamics simulations by DeAngelis et al. show that the binding occurs through three carboxylic acid groups and that the protons initially carried by the N719 dye are transferred to the semiconductor surface [61]. After covalent attachment to the TiO_2_ surface, the formed dipole might turn compared to the dipole of the free molecule in vacuum. After N719 has been chemisorbed onto TiO_2_, the surface is protonated and possesses a dipole pointing from the TiO_2_ surface to the negative net charge (isothiocyanato-group,-NCS) as shown in Figure 8 [38]. As a consequence the local vacuum level is bent upwards and the work function is increased compared with the bare TiO_2_ surface. For the nanoporous TiO_2_ surface sensitized with the Cu(I)-containing dye, a negative surface dipole pointing away from the surface leads to a decrease in work function. The copper(I) dye is a monocation in its fully protonated state, assuming that the phosphonic acid functionalities are fully protonated.

To quantify the surface dipole, Natan et al. proposed a plate capacitor model, in which the adsorbed molecules are regarded as point dipoles [62]:

[4]
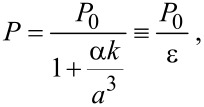


where *P* is the surface dipole moment, *P*_0_ is the dipole moment of the free molecule in vacuum, *k* is a geometric correction factor and *a* is the distance between two dipoles. The change in work function, ΔΦ*_S_*, is related to the surface dipole through the Helmholtz equation:

[5]
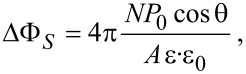


where (*N*/*A*) is the number of dipoles/molecules per surface area, ε = (*P*_0_/*P*) is the effective dielectric constant of a molecular monolayer and ε_0_ is the permittivity in vacuum. The dipole layer is oriented at an angle, θ, relative to the surface plane normal. Due to the curved surface geometry of nc-TiO_2_, the mean dipole in the *z*-direction is reduced (Figure 8).

A surface coverage of *N*/*A* = 1/4 molecules/nm^2^ is a reasonable value found by Ikeda et al. by AFM measurements of N3 (N719 is the salt of N3) adsorbed on rutile TiO_2_ in ultrahigh vacuum [42]. Using Equation 5 with θ = 0° and measured work-function shifts of ΔΦ = −180 ± 40 mV for the Cu(I) dye and ΔΦ = 150 ± 40 mV for N719 results in 6.3 ± 1.5 D and 5.3 ± 2 D with opposite directions, respectively. The latter value is in the same range as predicted by DFT calculations for N719 [38] and N3 [42,63] adsorbed on anatase plane-surface. However, for a complete DSC device the surface dipole may change due to screening by the surrounding electrolyte [64].

**Figure 9:**
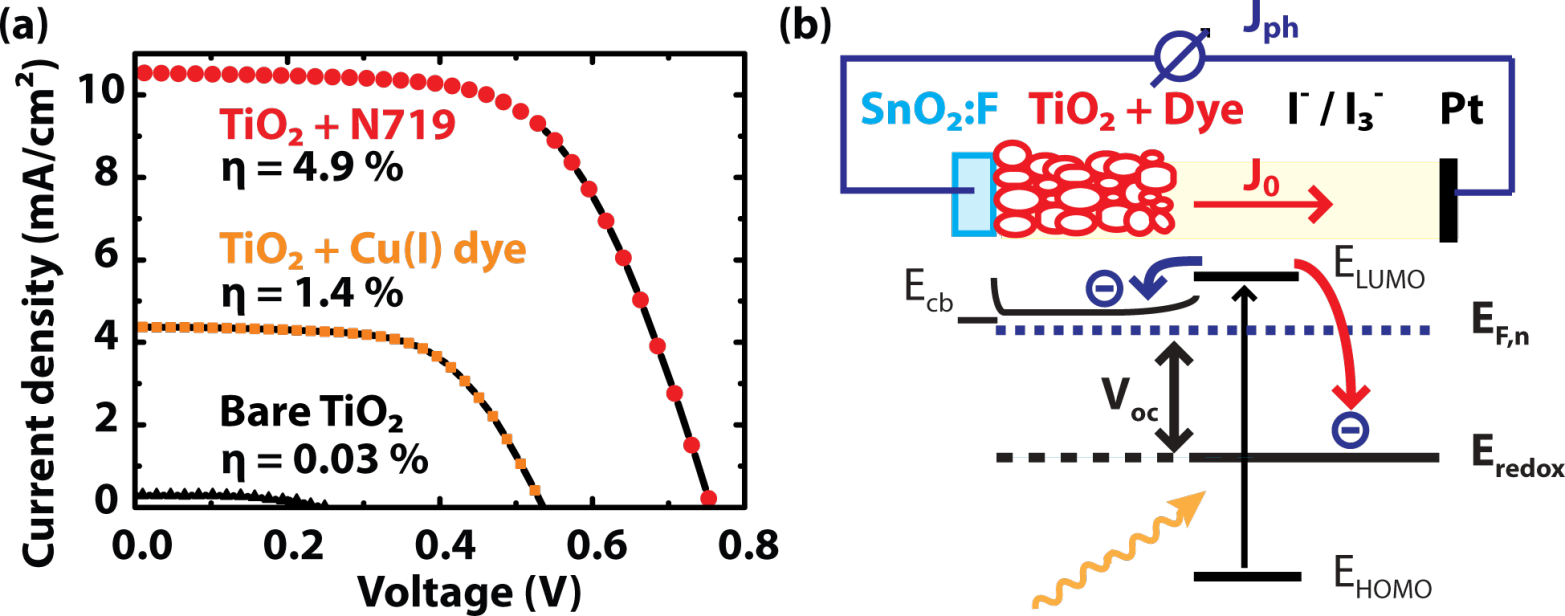
(a) *I*–*V* curves for a bare TiO_2_ solar cell and DSCs sensitized with Cu(I) dye and N719. (b) A schematic band diagram for a DSC under light excitation of the dye. The desired forward reaction (blue arrow), i.e., electron transfer from *E*_LUMO_ into the conduction band, *E*_cb_ of TiO_2_, is accompanied by a backward electron injection (red arrow) from *E*_LUMO_ into *E*_redox_.

Figure 9a depicts the *I*–*V* characteristics for three different DSCs, a bare TiO_2_ solar cell with electrolyte and DSCs sensitized with a Cu(I)-dye or N719, respectively. The parameters of the solar cell were extracted from the *I*–*V* data and are summarized in Table 1. A DSC sensitized with N719 is 3 times more efficient than the Cu-sensitized solar cell. We focus on the difference in the open-circuit voltages of −220 ± 20 mV between these DSCs. The deviation of the open-circuit voltages is in the range of the measured difference for the surface dipoles (350 ± 40 mV) formed by N719 and the copper(I) dye. The two sensitizers possess oppositely directed dipole moments.

**Table 1:** *I*–*V*-characteristic values of a DSC sensitized with N719 or the copper(I)-based dye.

dye	*J*_ph_ [mA/cm^2^]	*V*_oc_ [V]	FF	dipole direction	η [%]

Cu(I) dye	4	0.53	0.60	↑	1.4
N719	10	0.75	0.65	↓	4.9

Pandey et al. have investigated the surface dipoles of organic dye molecules adsorbed on TiO_2_ using KPFM [40]. They observed a decrease of *V*_oc_ for DSCs sensitized with dye molecules, which lead to a more positive surface potential. De Angelis et al. found that the adsorption geometry of the sensitizer on the TiO_2_ surface has a significant influence on *V*_oc_. The desired forward reaction, i.e., electron injection from *E*_LUMO_ into *E*_cb_ of TiO_2_ is accompanied by a backward electron injection from *E*_LUMO_ into *E*_redox_ (Figure 9b). This backward reaction is affected by the surface dipole of the adsorbed sensitizer [64]. Regarding the general expression for the open-circuit voltage (Equation 3), it is the reverse saturation current density, *J*_0_, and the conduction band minimum, *E*_cb_, that are influenced by the surface dipole and finally affect *V*_oc_:

[3]
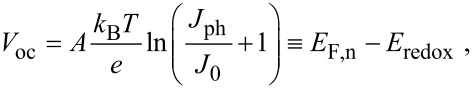


wherein *A* is the correction factor and *J*_ph_ the photocurrent density. A performance comparison of DSCs sensitized with N719 and the copper(I) dye is shown in Table 1.

## Conclusion

Microscopic surface photovoltage and work-function measurements were performed on bare and dye-sensitized TiO_2_ photoelectrodes using Kelvin probe force microscopy. Compared to a bare TiO_2_ layer, the surface potential is about 150 mV higher for an N719 sensitized TiO_2_ photoelectrode and about 180 mV lower for Cu-dye sensitized TiO_2_ resulting in a 200 mV higher open-circuit voltage (*V*_oc_) for a complete N719 DSC. We conclude that the surface dipole orientation is inverted for the two dyes and the *V*_oc_ of a complete DSC increases with a higher surface potential. Consequently, we assume that the detected microscopic surface potential drops/inhomogeneities on both bare and sensitized TiO_2_ photoelectrodes lead to a lower *V*_oc_ and efficiency of the solar cell. The bandgap of 3.2 eV for anatase nanocrystalline TiO_2_ particles was determined by SPV spectroscopy on different locations. It is constant throughout the TiO_2_ layer and in agreement with literature values for bulk anatase. The measured SPV under sub-bandgap illumination is formed at the SnO_2_:F/TiO_2_ interface due to the presence of a built-in electric field. According to our results, the interface barrier is around 250 mV. Its influence on the DSC performance is not resolved. In the case of dye-sensitized photoelectrodes, three different mechanisms contribute to the measured SPV. First, there is a contribution from the SnO_2_:F/TiO_2_ interface, which forms a heterojunction. The band diagram at this heterojunction is still unclear; however, the influence on our measurements is expected to be negligible, and thus, we decided not to include it in this paper. Secondly, there is an SPV contribution from the nc-TiO_2_ layer itself, and third, there is a reversible photochemical reaction of the dye molecule, which donates electrons under illumination and hence changes the surface potential. To our best knowledge, the measured SPV is not understood well enough to represent it generally in a band diagram without making major assumptions. Further studies are needed to clarify this point.

## Experimental

### Preparation of the photoanode

A glass substrate coated with SnO_2_:F (TCO 22-7, Solaronix) was cleaned in an ultrasonic bath successively with acetone, ethanol, and distilled water and subsequently treated with UV/ozone for 18 min (Model 42-220, Jelight Company). To study the SPV without a contribution from the SnO_2_:F/TiO_2_ interface, TiO_2_ layers were also prepared on uncoated glass substrates. A colloidal TiO_2_ solution (Ti-Nanoxide T, Solaronix) consisting of anatase nanoparticles (≈20 nm) was deposited by doctor blading. A mask with an area of 25 mm^2^ was defined with scotch tape. The TiO_2_ layer was sintered with a heating plate for 30 min at 500 °C resulting in a layer thickness of about 10 μm.

For the preparation of sensitized TiO_2_, the photoanode was immersed in a sensitizer solution for 24 h after cooling it to 80 °C. The ruthenium dye solution consisted of a 1 mM ethanol solution of standard dye N719. The nc-TiO_2_ was sensitized with the heteroleptic copper(I) dye shown in Figure 3 by using the procedure previously described, which involves an in situ assembly of the dye starting with the adsorbed anchoring ligand and the hexafluoridophosphate salt of the homoleptic complex [Cu(6-(2-thienyl)-2,2′-bipyridine)_2_]^+^ [43]. In order to remove weakly adsorbed contaminants, the sensitized TiO_2_ was rinsed with ethanol and dried under nitrogen.

### Kelvin probe force microscopy

**Figure 10:**
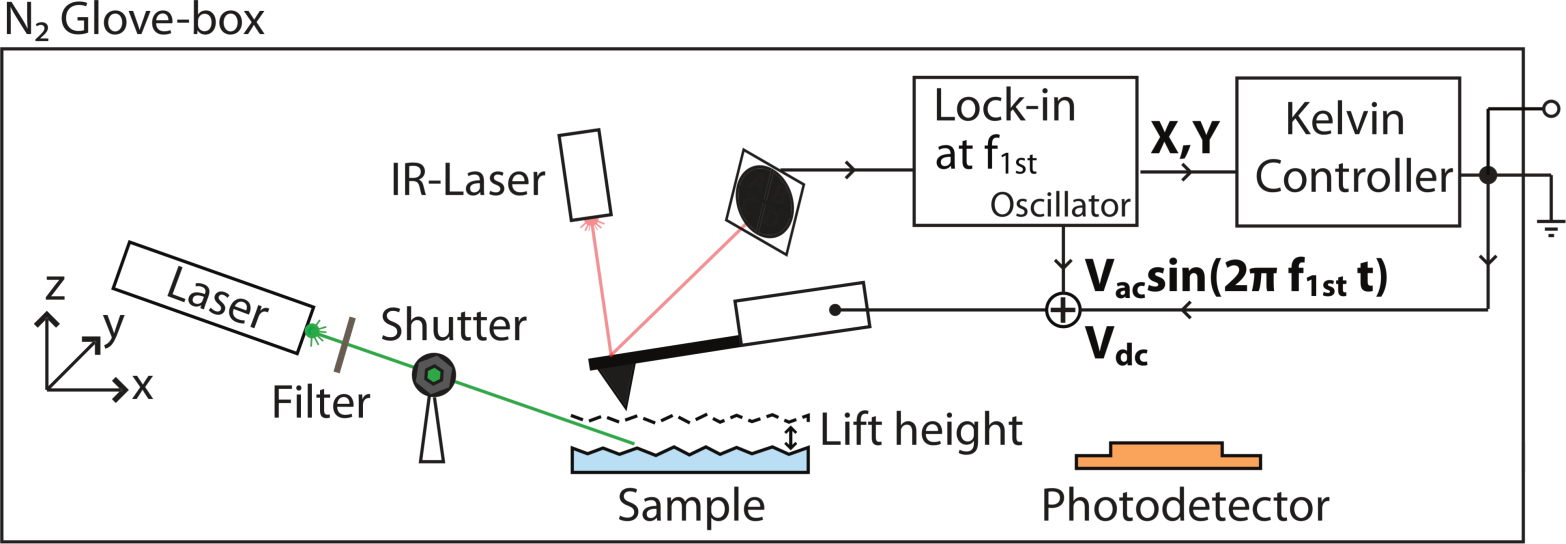
Lift-mode KPFM setup inside a nitrogen glove box in combination with a tunable illumination system for microscopic SPV measurements. The CPD was nullified by an applied dc voltage between tip and sample.

AFM measurements were carried out inside a glove box (labmaster 130, mBraun) maintaining a dry nitrogen atmosphere (<1 ppm H_2_O and <10 ppm O_2_) on a commercial microscope (Solver PH-47, NT-MDT). Amplitude modulation (AM) KPFM was conducted with a two-scan method (lift mode) meaning that the topography and CPD were measured separately (Figure 10). During the first line scan the topography was determined in tapping mode AFM using a bias voltage of *V*_dc_ = 0 V applied to the tip. The cantilever oscillation amplitude was kept constant by a feedback controller at a setpoint, *A*_sp_, of 20 nm rms that was pre adjusted to 75% of the free vibrational amplitude, *A*_0_ = 27 nm rms. The second scan was performed 20 nm above the previously obtained surface profile on the same scan line. While the sample was grounded, the dc and ac voltage were applied to the cantilever, *V*_ac_ = 2 V at a frequency equal to its first resonance, *f*_1st_. The X-component, X = A · cos(θ), of the electrically induced oscillation signal, which is proportional to the electrostatic force, *F*_es_, was phase-adjusted and retrieved with a lock-in amplifier and subsequently nullified by the applied dc voltage in the Kelvin feedback. Cantilever bending and vibration were optically detected with an infrared laser (λ = 1300 nm, 1 mW, spot size 50 × 50 μm^2^) and a four-quadrant photodetector. To deduce the sample work function and monitor the integrity of the tip, a highly ordered pyrolytic graphite (HOPG) reference sample was measured before and after each measured specimen. The HOPG reference with a known work function of 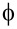 = 4.6 eV [65] was stored inside the glove box.

### Microscopic photovoltage determination

A UV-lamp (360 nm, L18W/73, Osram) was used for super-bandgap illumination. Trap and interface states were investigated under sub-bandgap illumination (λ > 385 nm) with LEDs (405 nm, 470 nm, 530 nm, 590 nm, Mightex Systems) or solid state lasers each mounted on a x-y-z positioning stage. A red laser (660 nm, 60 mW, LPM660-60C, Newport), a green laser (532 nm, 4 mW, Alpec), a violet laser (408 nm, 2.5 mW, Power Technology) and LEDs were applied for illumination from above at an angle of 10° with respect to the sample plane. The laser illumination was switched with a shutter (T132, Uniblitz). Laser light intensity was gradually reduced with different neutral density (ND) filters. Incident light intensity was measured at the sample location with a calibrated silicon photodetector (PD300-UV-SH, Ophir) and a power meter head (AN/2, Ophir). SPV spectroscopy was conducted on a commercial AFM (Dimension 3100, Bruker). The cantilever vibration was optically detected with a red laser (670 nm, 1 mW, spot size ≈ 40 × 40 μm^2^), which partially hit the sample surface with an intensity of ≈100 μW. In order to avoid background illumination by the red laser, the spot was positioned on the cantilever center. The light of a xenon-arc lamp (300 W, model 6258, Newport) was focused onto the entrance slit of a grating monochromator (MS257, Newport) with a resolution of 5 nm in the range between 350–700 nm. The monochromatic light was coupled into an optical light guide (3 mm, 1.83 m, *N*_A_ = 0.55, Edmund optics) which was connected to an optical microscope built-in within (inside) the AFM. Finally, the outcoming light of the optical microscope was focused onto the sample surface with a spot diameter of about 500 μm and a depth of focus of about 10 μm.

### Photovoltaic characterisation of DSCs

*I*–*V*-curves were measured under an AM1.5G solar simulator (ABET) with an intensity of 100 mW/cm^2^ and a controlled temperature of 25 °C. The voltage sweep was performed in 6 mV steps with a sampling rate of 1 Hz.

## References

[1] O’Regan B, Grätzel M (1991). Nature.

[2] Green M A, Emery K, Hishikawa Y, Warta W, Dunlop E D (2012). Prog Photovoltaics.

[3] Wang H, Liu M, Yan C, Bell J (2012). Beilstein J Nanotechnol.

[4] Motta N (2012). Beilstein J Nanotechnol.

[5] Kaneko M, Ueno H, Nemoto J (2011). Beilstein J Nanotechnol.

[6] Bora T, Kyaw H H, Sarkar S, Pal S K, Dutta J (2011). Beilstein J Nanotechnol.

[7] Wang M, Chamberland N, Breau L, Moser J-E, Humphry-Baker R, Marsan B, Zakeeruddin S M, Grätzel M (2010). Nat Chem.

[8] Wang M, Xu M, Shi D, Li R, Gao F, Zhang G, Yi Z, Humphry-Baker R, Wang P, Zakeeruddin S M (2008). Adv Mater.

[9] Alonso-Vante N, Nierengarten J, Sauvage J (1994). J Chem Soc, Dalton Trans.

[10] Constable E C, Redondo Hernandez A, Housecroft C E, Neuburger M, Schaffner S (2009). Dalton Trans.

[11] Bozic-Weber B, Constable E C, Hostettler N, Housecroft C E, Schmitt R, Schönhofer E (2012). Chem Commun.

[12] Bozic-Weber B, Brauchli S Y, Constable E C, Fürer S O, Housecroft C E, Wright I A (2013). Phys Chem Chem Phys.

[13] Bozic-Weber B, Constable E C, Housecroft C E (2013). Coord Chem Rev.

[14] Yella A, Lee H-W, Tsao H N, Yi C, Chandiran A K, Nazeeruddin M K, Diau E W-G, Yeh C-Y, Zakeeruddin S M, Grätzel M (2011). Science.

[15] Nicholson P G, Castro F A (2010). Nanotechnology.

[16] Shen Q, Ogomi Y, Park B-w, Inoue T, Pandey S S, Miyamoto A, Fujita S, Katayama K, Toyoda T, Hayase S (2012). Phys Chem Chem Phys.

[17] Graaf H, Maedler C, Kehr M, Baumgaertel T, Oekermann T (2009). Phys Status Solidi A.

[18] Kronik L, Shapira Y (1999). Surf Sci Rep.

[19] Kronik L, Shapira Y (2001). Surf Interface Anal.

[20] Schroder D K (2001). Meas Sci Technol.

[21] Cahen D, Hodes G, Graetzel M, Guillemoles J F, Riess I (2000). J Phys Chem B.

[22] Lenzmann F, Krueger J, Burnside S, Brooks K, Grätzel M, Gal D, Rühle S, Cahen D (2001). J Phys Chem B.

[23] Beranek R, Neumann B, Sakthivel S, Janczarek M, Dittrich T, Tributsch H, Kisch H (2007). Chem Phys.

[24] Yang J, Warren D S, Gordon K C, McQuillan A J (2007). J Appl Phys.

[25] Rühle S, Greenshtein M, Chen S G, Merson A, Pizem H, Sukenik C S, Cahen D, Zaban A (2005). J Phys Chem B.

[26] Duzhko V, Timoshenko V Y, Koch F, Dittrich T (2001). Phys Rev B.

[27] Rothschild A, Levakov A, Shapira Y, Ashkenasy N, Komem Y (2003). Surf Sci.

[28] Liu Y, Scully S R, McGehee M D, Liu J, Luscombe C K, Fréchet J M J, Shaheen S E, Ginley D S (2006). J Phys Chem B.

[29] Warren D S, Shapira Y, Kisch H, McQuillan A J (2007). J Phys Chem C.

[30] Weaver J M R, Wickramasinghe H K (1991). J Vac Sci Technol, B.

[31] Saraf S, Shikler R, Yang J, Rosenwaks Y (2002). Appl Phys Lett.

[32] Streicher F, Sadewasser S, Lux-Steiner M C (2009). Rev Sci Instrum.

[33] Nonnenmacher M, O'Boyle M P, Wickramasinghe H K (1991). Appl Phys Lett.

[34] Glatzel T, Sadewasser S, Glatzel T (2011). Measuring Atomic-Scale Variations of the Electrostatic Force. Kelvin Probe Force Microscopy.

[35] Milde P, Zerweck U, Eng L M, Abel M, Giovanelli L, Nony L, Mossoyan M, Porte L, Loppacher C (2008). Nanotechnology.

[36] Glatzel T, Zimmerli L, Koch S, Kawai S, Meyer E (2009). Appl Phys Lett.

[37] Kittelmann M, Rahe P, Gourdon A, Kühnle A (2012). ACS Nano.

[38] De Angelis F, Fantacci S, Selloni A, Grätzel M, Nazeeruddin M K (2007). Nano Lett.

[39] Sakaguchi S, Pandey S S, Okada K, Yamaguchi Y, Hayase S (2008). Appl Phys Expr.

[40] Pandey S S, Sakaguchi S, Yamaguchi Y, Hayase S (2010). Org Electron.

[41] Hiehata K, Sasahara A, Onishi H (2007). Nanotechnology.

[42] Ikeda M, Koide N, Han L, Sasahara A, Onishi H (2008). J Phys Chem C.

[43] Bozic-Weber B, Constable E C, Housecroft C E, Kopecky P, Neuburger M, Zampese J A (2011). Dalton Trans.

[44] Tan M, Wang G, Zhang L (1996). J Appl Phys.

[45] Łagowski J, Balestra C L, Gatos H C (1972). Surf Sci.

[46] Schwarzburg K, Willig F (1991). Appl Phys Lett.

[47] Moss T S (1959). Optical properties of semi-conductors.

[48] Kavan L (2012). Chem Rec.

[49] Howe R F, Grätzel M (1985). J Phys Chem.

[50] Peter L M (2007). Phys Chem Chem Phys.

[51] Levy B, Liu W, Gilbert S E (1997). J Phys Chem B.

[52] Kron G, Rau U, Werner J H (2003). J Phys Chem B.

[53] Kronik L, Ashkenasy N, Leibovitch M, Fefer E, Shapira Y, Gorer S, Hodes G (1998). J Electrochem Soc.

[54] Dittrich T, Duzhko V, Koch F, Kytin V, Rappich J (2002). Phys Rev B.

[55] Dittrich T (2000). Phys Status Solidi A.

[56] Bonhôte P, Moser J-E, Humphry-Baker R, Vlachopoulos N, Zakeeruddin S M, Walder L, Grätzel M (1999). J Am Chem Soc.

[57] Nelson J (1999). Phys Rev B.

[58] Timoshenko V Y, Kashkarov P K, Matveeva A B, Konstantinova E A, Flietner H, Dittrich T (1996). Thin Solid Films.

[59] Cohen R, Bastide S, Cahen D, Libman J, Shanzer A, Rosenwaks Y (1998). Opt Mater.

[60] Lee K E, Gomez M A, Elouatik S, Demopoulos G P (2010). Langmuir.

[61] De Angelis F, Fantacci S, Selloni A, Nazeeruddin M K, Grätzel M (2010). J Phys Chem C.

[62] Natan A, Zidon Y, Shapira Y, Kronik L (2006). Phys Rev B.

[63] Fantacci S, De Angelis F, Selloni A (2003). J Am Chem Soc.

[64] Miyashita M, Sunahara K, Nishikawa T, Uemura Y, Koumura N, Hara K, Mori A, Abe T, Suzuki E, Mori S (2008). J Am Chem Soc.

[65] Sommerhalter C, Matthes T W, Glatzel T, Jäger-Waldau A, Lux-Steiner M C (1999). Appl Phys Lett.

